# Collaborative Decision-Making in Implant Abutment Selection: Perspectives of Dentists and Dental Technicians in a Cross-Sectional Study

**DOI:** 10.3390/healthcare14172793

**Published:** 2026-09-01

**Authors:** Omir Aldowah

**Affiliations:** 1 Department of Prosthetic Dental Science, Najran University, Najran 61441, Saudi Arabia; osaldowah@nu.edu.sa; 2 Research Medical Centre, Najran University, Najran 61441, Saudi Arabia

**Keywords:** collaborative decision-making, dental implants, dental technicians, dentist–technician communication, implant abutment selection, implant prosthodontics

## Abstract

**Aim:** To investigate implant abutment selection practices among dentists and dental laboratory technicians, with particular emphasis on factors influencing abutment selection, communication, collaborative decision-making, and reported implant abutment-related complications. **Methods:** A cross-sectional questionnaire survey was conducted among dentists involved in implant prosthodontics and dental laboratory technicians. The questionnaire assessed factors influencing implant abutment selection, communication practices, collaborative decision-making, and self-reported complications. Descriptive statistics were calculated, and associations between categorical variables were evaluated using the Fisher–Freeman–Halton exact test when appropriate. **Results:** A total of 144 eligible participants (101 dentists and 43 dental technicians) were included. Among dentists, the most frequently reported factors influencing implant abutment selection were implant position and angulation (97.0%), inter-occlusal space (82.2%), aesthetic requirements (78.2%), and soft tissue thickness and contour (78.2%). Most dentists reported providing clear written or verbal instructions to dental technicians, while most technicians reported supporting dentists in abutment selection and initiating communication when the selected abutment was considered inappropriate. No statistically significant associations were observed between communication-related variables and dentists’ reported clinical complications or technicians’ perceptions of work-related problems due to poor communication (*p* = 0.870 and *p* = 0.420, respectively). However, technicians who preferred collaborative decision-making were significantly more likely to report taking the initiative to communicate with dentists regarding inappropriate abutment selection (Fisher–Freeman–Halton exact test, *p* = 0.040). **Conclusions:** Selection of implant abutment was primarily directed by both clinical and prosthetic considerations. Dentists and technicians preferred collaborative decision-making, even though no statistically significant association was noticed between communication and self-reported complications. Further studies with greater representative samples and objective clinical outcomes are necessary.

## 1. Introduction

Implant-supported prostheses are an increasingly established therapeutic option in dental practice for restoring function to both partially and fully dentate patients [1]. This is largely due to the high level of predictability that exists for the long-term survival of these implants as well as the excellent results that can be expected with regard to aesthetics and function [2]. Implant abutments act as a bridge between dental implants and the final restoration. Consequently, they are integral to maintaining the mechanical integrity of implant supported dentures [3]. The choice of appropriate abutment has significant implications for the overall success of the restoration; specifically, it relates directly to the mechanical retention of the abutment, the health of the surrounding soft tissues, the emergence profile, cleaning efficacy, the distribution of masticatory forces (occlusion), and ultimately whether or not the implant will remain functional over time [4].

The selection of the proper implant abutment is clinically a multifaceted choice which will require the evaluation of several factors related to both the patient and the prosthesis. Factors such as the location and angle of the implants, amount of occlusal space available between opposing teeth, the thickness and form of the surrounding soft-tissue as well as the type of dental restoration planned; restorative material, aesthetics desired by the patient and ease of access for oral hygiene are all important [5]. If there is some compromise in the placement of an implant due to either anatomical limitations or limited space for a restorative reconstruction, clinicians may elect to utilize custom or angled abutments [6]. This will allow them to create optimal prosthetic contours while also reducing potential mechanical and biological problems. Consequently, the selection of an appropriate implant abutment should be based on both biomechanical/prosthetic principles, as well as other practical variables (e.g., availability, cost) that impact a practitioner’s decision-making [7]. While practical issues may significantly affect the final choice of which implants to use in each case, they are generally secondary to the biological and biomechanical aspects of abutments used with dental implants [8].

The success of implant rehabilitation is dependent upon the collaboration of both clinical professionals and dental laboratory technicians from the time a clinician develops a treatment plan for an individual with implants through to when the prosthesis has been fabricated by the technician and delivered back to the clinician [9]. Clinicians are able to combine the specific needs of each patient (biological, functional and aesthetic) in developing their final treatment plan. The contribution of the dental technician can be considered as having technical knowledge of all aspects of implant components, restorative materials, digital workflows and fabrication techniques [10]. Therefore, it is necessary for communication among these professional groups. This is to allow that a prescription from the laboratory reflects the clinical needs as well as identify all possible issues related to restorations prior to fabrication of prosthesis. Previous studies have established the importance of dentist-technician communication when attempting to achieve predictable prosthodontic results and prevent laboratory errors [11,12].

Despite recognized benefits, communication between dental clinics and laboratories is not always very good. Poor communication can contribute to planning errors for treatment, fabrication of prostheses and selection of abutments for implants. Combined with other clinical, surgical and patient factors this can indirectly increase likelihood of technical and biological complications [9,13]. Prior research has documented that less than half of all dental technicians felt they were inadequately engaged by their respective dental teams, also, roughly a third of these same dental technicians felt that dentists did not welcome or encourage communications from them [13]. Therefore, an urgent need exists to improve collaboration and communication among professionals involved in the restorative process.

Most of the past studies investigated dentist-technician communication with respect to laboratory prescriptions, work authorizations, fabrication techniques or general consultative practices [12,14,15]. In addition, much of the research conducted in the area of implant prosthodontics has been centred around prosthetic failures, treatment modalities or clinical results as opposed to the decision-making process involved in selecting an appropriate implant abutment [16,17,18].

Though the impact of clinical and mechanical factors of selecting implant abutment were discussed [5,7], attention was noticeably less directed toward how this selection decision is made between dentists and dental technicians [13,14]. Several studies stated variances of the planning of dentists and technicians in professional perceptions, communication practices, and the personal contributions [13,14,19].

However, there are few studies that have investigated the factors affecting the choice of an implant abutment; the communication strategies used in dentist-dental technician teams; and the views held by dentists and dental technicians concerning collaboration as a part of making decisions relating to implant rehabilitation [19]. Addressing this gap may expand understanding of existing interdisciplinary practices. It also may identify weaknesses in which clinical-to-laboratory communication can be improved and how implant abutment selection can be applied.

Therefore, this cross-sectional study aimed to investigate implant abutment selection practices among dentists and dental laboratory technicians. Specifically, the study examined the factors influencing implant abutment selection, communication practices between dentists and dental technicians, collaborative decision-making, and the association between communication-related variables and reported implant abutment-related complications.

## 2. Materials and Methods

### 2.1. Study Design and Ethical Approval

This cross-sectional study was conducted using two structured electronic questionnaires, one designed for dentists and the other for dental technicians. This study was approved by the Research Ethics Committee of Najran University, Saudi Arabia. The Approval No. is 202509-076-035361-079258. This study was performed in accordance with the ethical principles of the Declaration of Helsinki and the regulations of the National Committee for Bioethics (NCBE), King Abdulaziz City for Science and Technology (KACST), Saudi Arabia.

### 2.2. Sample Size Calculation

An a priori sample size calculation using G*Power software (version 3.1.9.7; Heinrich Heine University, Düsseldorf, Germany) indicated that a minimum of 143 participants would provide 80% statistical power to detect a medium effect size (w = 0.30) using the chi-square test with a significance level of α = 0.05. The medium effect size was chosen based on Cohen’s conventional effect-size benchmarks due to the lack of a sufficiently similar previous study to provide a study-specific effect estimate. The study sample comprised 144 eligible individuals (101 dentists and 43 dental technicians), exceeding the intended minimum overall sample size. The a priori calculation was formulated based on the total study sample and was not precisely powered for subgroup analyses within the dental technician group.

### 2.3. Participants and Recruitment

The survey was conducted between Jan 2026 and April 2026 in Saudi Arabia. A convenience sampling strategy was used to recruit licensed dentists involved in implant prosthodontic treatment and dental technicians who participated in the fabrication of implant-supported prostheses. Participants were recruited through professional networks, academic institutions, hospitals, private dental centers, and professional communication groups using Google Forms. Participation was voluntary and anonymous, and the introductory page described the study objectives, participant rights, and informed consent requirements before access to the questionnaire was granted.

Eligible participants included licensed dentists and dental technicians who were actively involved in the restoration of implant-supported prostheses. Dental trainees or residents were eligible only if they were directly involved in implant prosthodontic treatment as part of their clinical training. No minimum years of implant prosthodontic experience were required. The questionnaires were administered in English for the dentists’ and in Arabic for the dental technicians. To minimize duplicate submissions, the survey platform was configured to accept only one response per Google account to restrict duplicate submissions, and participants were instructed to complete the survey only once.

Because the survey link was disseminated through multiple professional networks, the exact number of individuals who received the invitation could not be determined; therefore, a response rate could not be calculated. No reminder messages were distributed to complete the survey. Questionnaires without informed consent were excluded from the analysis. Participants who indicated that they were not involved in restoring implant-supported prostheses were excluded from implant-specific analyses through conditional branching. Incomplete questionnaires were included only for completed items, and percentages were calculated using the number of valid responses for each questionnaire item.

A total of 106 dentists accessed the survey, of whom 103 provided informed consent. Among the consenting dentists, two reported that they did not restore implant-supported prostheses and were therefore excluded from implant-specific analyses. Consequently, implant-related analyses were based on 101 eligible dentists. All 43 participating dental technicians met the eligibility criteria and were included in the analyses. Because the questionnaire employed conditional branching and some participants did not answer every applicable question, the valid sample size varied across individual analyses.

### 2.4. Questionnaire Development and Data Collection

Two structured electronic questionnaires were developed specifically for this study based on a review of the published literature on implant prosthodontics, implant abutment selection, and dentist–technician communication. The initial questionnaire items were generated from previous study [20] and adapted to address the objectives of the present investigation. The draft questionnaires were reviewed by a panel of experts (two dentists and one dental technician) in prosthodontics and implant dentistry to assess content relevance, clarity, and comprehensiveness. Following expert review, minor modifications were made to improve wording and clarity.

The questionnaires were subsequently pilot tested with 10 dentists and dental technicians to evaluate readability, comprehension, and completion time. Feedback obtained during pilot testing resulted in minor editorial revisions, and pilot responses were not included in the final analysis. The questionnaire for dental technicians was translated in Arabic using forward and backward translation procedures to ensure linguistic equivalence.

The final questionnaires included items on participants’ demographic and professional characteristics, communication practices between dentists and dental technicians, factors influencing implant abutment selection, sources of professional knowledge, reported implant abutment-related complications, collaborative decision-making, and perceived responsibility for complications. Communication practices and perceptions were primarily assessed using Likert-scale questions, whereas factors influencing implant abutment selection, sources of knowledge, complications, and responsibility were assessed using multiple-response questions.

The questionnaires were designed as structured survey instruments to address several different domains rather than as psychometric scales proposed to create a compound score for a definite latent construct. Therefore, an overall internal consistency coefficient (Cronbach’s alpha) and construct validity analysis were not applicable to the whole surveys. Also, quantitative assessment using a Content Validity Index and test–retest reliability was not conducted.

### 2.5. Statistical Analysis

Statistical analyses were performed using IBM SPSS Statistics version 26 (IBM Corp., Armonk, NY, USA). Descriptive statistics were used to summarize participant characteristics and questionnaire responses and are presented as frequencies and percentages for categorical variables. Owing to conditional branching within the questionnaire and occasional item non-response, the valid sample size varied across analyses, and percentages were calculated using the number of valid responses for each questionnaire item. Multiple-response questions were analyzed descriptively, with percentages calculated according to the number of respondents selecting each option.

Associations between categorical variables were initially assessed using the Pearson chi-square test. When the assumptions of the Pearson chi-square test were violated because of sparse expected cell counts, the Fisher–Freeman–Halton exact test with Monte Carlo estimation was used. Statistical significance was set at *p* < 0.05. An exploratory multivariable binary logistic regression was executed among clinicians to find associated factors of reported implant abutment-related complications. Responses were classified into two groups: agree/strongly agree versus other responses. The model comprised professional experience, specialty, postgraduate education area, workplace sector, and communication frequency with dental technicians. Odds ratios (ORs) with 95% confidence intervals (CIs) were testified.

## 3. Results

**Figure 1:**
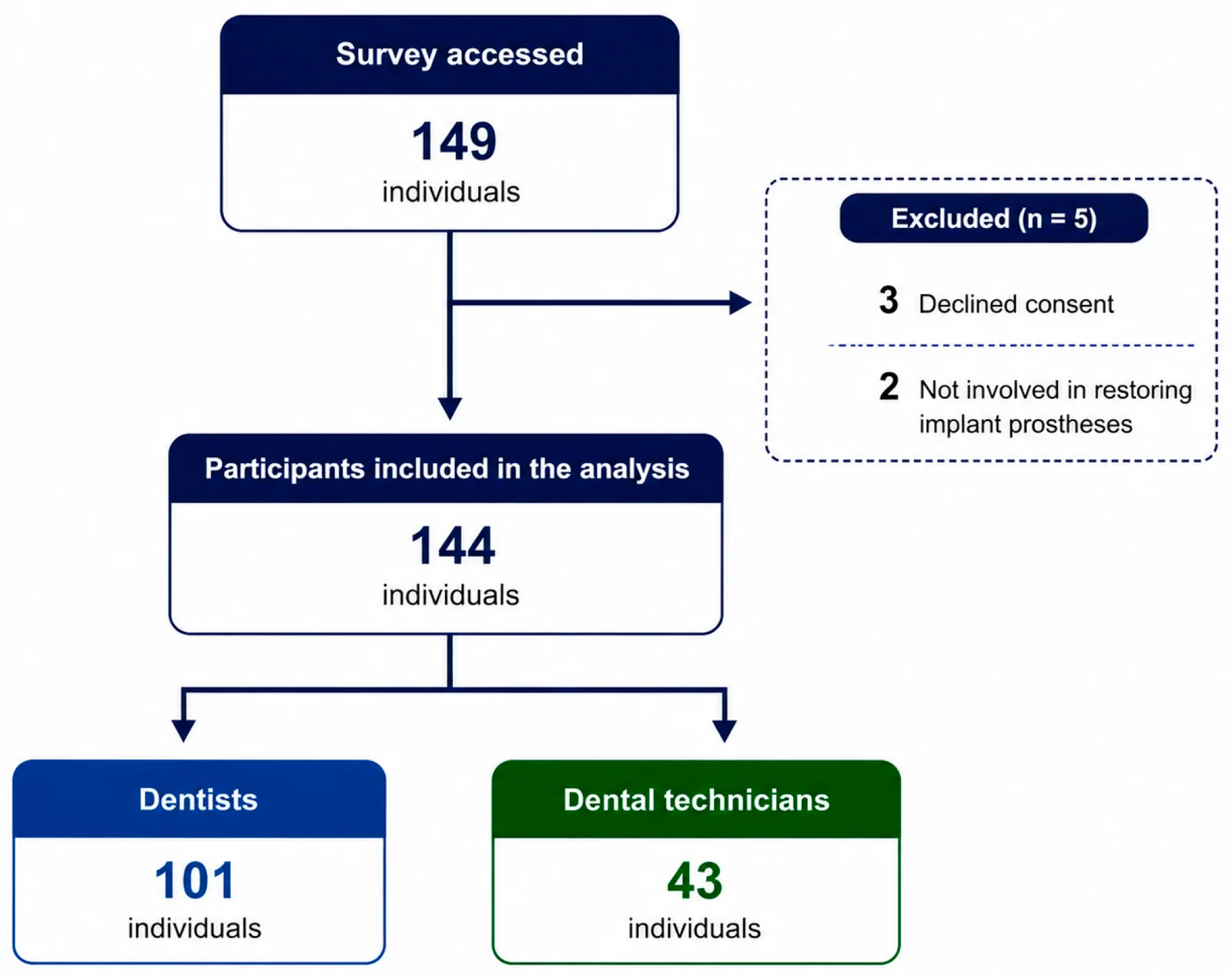
Participant flow diagram of the study.

A total of 149 participants accessed the survey, including 106 dentists and 43 dental technicians. Among the dentists, 103 provided informed consent, whereas three individuals who did not consent were excluded from all analyses. Of the consenting dentists, two reported that they were not involved in restoring implant-supported prostheses and were therefore excluded from implant-specific analyses, resulting in 101 eligible dentists for implant-related descriptive and inferential analyses. All 43 dental technicians met the eligibility criteria and were included in the analyses. Because the survey employed conditional branching and some participants did not answer every applicable question, the valid sample size varied across individual analyses. The denominator used for each analysis is reported in the corresponding tables. The participant recruitment, eligibility assessment, exclusions, and final analytical samples for both professional groups are summarized in Figure 1.

Figure 1 illustrating participant recruitment, eligibility assessment, and inclusion in the analyses. Of the 106 dentists who accessed the survey, 103 provided informed consent. Three individuals who did not provide consent were excluded from all analyses. Among the consenting dentists, two who reported not restoring implant-supported prostheses were excluded from implant-specific analyses, resulting in 101 eligible dentists. All 43 dental technicians who accessed the survey consented and met the eligibility criteria. Owing to conditional branching and item non-response, the valid sample size (N) varied across individual descriptive and inferential analyses. The descriptive characteristics and questionnaire responses of dentists and dental technicians are presented in Table 1 and Table 2.

Table 3 shows there was no statistically significant association between dentists’ provision of clear written or verbal instructions to the laboratory for implant abutments and experiencing clinical complications related to inappropriate implant abutment selection (Fisher–Freeman–Halton exact test = 7.439, Monte Carlo exact *p* = 0.870).

The descriptive distribution of factors influencing implant abutment selection based on dentists’ communication with dental technicians is illustrated in Supplementary Table S1.

Table 4 shows that there was no statistically significant association between dental technicians’ perception that dentists provide clear instructions regarding the type of abutment and their perception that problems occur due to poor communication with the dentist (Fisher–Freeman–Halton exact test = 16.462, Monte Carlo exact *p* = 0.420). Because the assumptions of the Pearson chi-square test were violated owing to sparse expected cell counts, the Fisher–Freeman–Halton exact test was used as the primary analysis.

Table 5 shows a statistically significant association between dental technicians’ preference for collaborative decision-making regarding implant abutment selection and their willingness to take the initiative to communicate with the dentist when the selected abutment was considered inappropriate (Fisher–Freeman–Halton exact test = 8.391, Monte Carlo exact *p* = 0.040). Because the assumptions of the Pearson chi-square test were violated owing to sparse expected cell counts, the Fisher–Freeman–Halton exact test was used as the primary analysis. These findings suggest that technicians who preferred collaborative decision-making were more likely to report taking the initiative to communicate with the dentist regarding abutment selection.

Regarding the multivariable analysis, none of the assessed professional or communicative factors were independently linked to reported implant abutment-related complications Table 6. Regular communication with dental technicians did not show a significant correlation with reported complications after controlling for dentists’ experience, specialities, postgraduate education, and work sector (OR = 0.73; 95% CI: 0.43–1.24; *p* = 0.242). The overall model was not statistically significant (*p* = 0.381).

## 4. Discussion

This study investigated the decision-making processes involved in implant abutment selection from the perspectives of both dentists and dental technicians. Particular emphasis was placed on the factors influencing abutment selection, communication between dentists and laboratory technicians, collaborative decision-making, and the occurrence of implant abutment-related complications. Overall, the findings indicate that implant abutment selection is a multifactorial process in which clinical, prosthetic, and aesthetic considerations play central roles. Furthermore, both dentists and dental technicians demonstrated a strong preference for collaborative decision-making during implant treatment planning. While communication was perceived as an important component of the restorative workflow, the present findings suggest that the communication-related variables evaluated in this study were not significantly associated with self-reported clinical complications related to implant abutment selection.

One of the principal findings of this study was the widespread support for interdisciplinary collaboration between dentists and dental technicians. Most technicians reported that they actively contribute to abutment selection and indicated that they communicate with dentists whenever they believe the selected abutment is inappropriate. Similarly, most dentists and technicians preferred that implant abutment decisions be made collaboratively rather than independently. Furthermore, a statistically significant association was observed between technicians’ preference for collaborative decision-making and their willingness to initiate communication with dentists regarding inappropriate abutment selection. This finding suggests that technicians who value collaborative treatment planning are more likely to actively participate in the decision-making process when potential restorative concerns are identified. These observations reinforce the interdisciplinary nature of implant dentistry and support the concept of prosthetically driven treatment planning, in which surgeons, restorative dentists, and dental technicians work together to achieve optimal functional and esthetic outcomes. Previous studies have similarly emphasized that successful implant rehabilitation depends on effective communication and collaboration throughout treatment planning, prosthesis fabrication, delivery, and long-term maintenance [9,21,22].

The descriptive findings also demonstrated that implant position and angulation, inter-occlusal space, aesthetic requirements, prosthesis type, and soft tissue thickness and contour were the factors most frequently considered by dentists during implant abutment selection. In contrast, cost, component availability, familiarity with a specific implant system, and recommendations from implant manufacturers were selected less frequently. Because participants were allowed to select multiple factors, these findings are descriptive and should not be interpreted as evidence of statistical associations. However, they indicate that clinical and prosthetic considerations were generally prioritized over logistical or commercial factors during treatment planning.

Among the factors influencing abutment selection, implant position and angulation appeared to be the most frequently considered. This observation is consistent with previous systematic reviews demonstrating that implant angulation influences prosthetic design, abutment selection, screw-access location, emergence profile, and load distribution [23]. When implant positioning is less than ideal, customized or angled abutments are frequently required to optimize prosthetic contours while minimizing biological and mechanical complications [7]. Likewise, inter-occlusal space was commonly considered during abutment selection, reflecting the importance of providing adequate restorative space for implant-supported prostheses. Insufficient restorative space has been associated with compromised prosthetic design, mechanical complications, and reduced aesthetic outcomes [24].

Soft tissue thickness and contour were also frequently considered during abutment selection. This finding agrees with previous evidence demonstrating that peri-implant soft tissue characteristics influence aesthetic integration, tissue stability, and long-term peri-implant health [25]. Appropriate abutment design and material selection therefore contribute not only to mechanical performance but also to the maintenance of favourable peri-implant soft tissue architecture. Similarly, aesthetic requirements were commonly identified as important considerations, reflecting increasing patient expectations regarding implant-supported restorations, particularly in the aesthetic zone. Previous investigations have shown that abutment material, emergence profile, and prosthetic contours significantly influence aesthetic outcomes and patient satisfaction [26].

Although communication was regarded as an essential component of implant treatment, the present study did not identify statistically significant associations between communication-related variables and reported clinical complications. Dentists who reported providing clear written or verbal laboratory instructions were not significantly less likely to report complications related to inappropriate implant abutment selection. Similarly, dental technicians’ perceptions that dentists provided clear instructions were not significantly associated with their reports of work-related problems caused by poor communication. These findings suggest that, although effective communication remains an essential aspect of interdisciplinary care, the occurrence of implant-related complications is influenced by multiple interacting factors beyond communication alone [27].

The absence of statistically significant associations between communication variables and clinical complications should not be interpreted as evidence that communication is unimportant. Implant complications arise from complex interactions among surgical, prosthetic, material, occlusal, and patient-related factors. Previous systematic reviews have demonstrated that mechanical complications such as screw loosening, screw fracture, prosthesis fracture, and component wear may occur despite appropriate treatment planning because of occlusal overload, implant positioning errors, prosthetic design limitations, material fatigue, or component-related factors [17,18,28]. Consequently, effective communication should be considered one element within a broader multidisciplinary approach to minimizing complications rather than an isolated determinant of clinical success.

A considerable proportion of participants reported experiencing clinical complications that they attributed to inappropriate implant abutment selection. Screw loosening was the most frequently reported complication, followed by implant-abutment misfit, screw fracture, occlusal discrepancies, prosthesis fracture or chipping, peri-implant mucositis, peri-implantitis, and abutment fracture. These findings are consistent with previous reports identifying screw-related complications as among the most common technical complications associated with implant-supported restorations [17,29]. Mechanical complications at the implant-abutment interface remain clinically important because they may compromise prosthesis stability, increase maintenance requirements, and contribute indirectly to biological complications through micromovement and plaque accumulation [30].

The relatively high frequency of implant-abutment misfit reported by participants is also noteworthy. Passive fit has long been considered essential for implant-supported prostheses because discrepancies at the implant-abutment interface increase mechanical stresses that may contribute to screw loosening, component fracture, and prosthetic failure [31]. Such discrepancies may result from impression inaccuracies, digital workflow limitations, laboratory fabrication errors, or inappropriate abutment selection. These findings further emphasize the importance of careful prosthetic planning and appropriate abutment selection throughout implant rehabilitation.

Biological complications, including peri-implant mucositis and peri-implantitis, were also reported, although less frequently than mechanical complications. Previous evidence indicates that prosthetic factors, including emergence profile, restoration contour, abutment material, and accessibility for oral hygiene, may influence peri-implant tissue health [32,33]. Therefore, implant abutment selection should balance mechanical stability, aesthetic requirements, and the maintenance of healthy peri-implant tissues to optimize long-term treatment outcomes.

Although multivariable analysis revealed that communication with technicians was not independently associated with reported implant abutment complications, communication with technicians may influence complications. Implant mechanical complications are multifactorial and may be affected by factors not evaluated in this study, including variety of implant systems, experience of professionals, digitalization of the lab. organization of the team, and clinical-laboratory workflow [5,9,17,34]. Additionally, the relatively low sample size and dependence on self-reported complications may have restricted the capability to identify such associations. So, statistical insignificance here should be interpreted with caution, and an independent effect of communication may remain as influential as reported previously by both dentists and technicians.

The lack of statistically significant associations should also be inferred in the perspective of statistical power. While the total sample met the sample-size criteria, the smaller dental technician subgroup (n = 43) may have limited the possibility to notice associations, especially in analyses involving multiple response levels. Consequently, Type II error cannot be omitted, and non-significant results should not be translated as proof of the absence of an underlying association.

### Limitations

This study has several limitations. First, the cross-sectional design allows the assessment of current practices and perceptions but does not permit causal inference. Second, the use of self-reported questionnaires introduces the possibility of recall bias and social desirability bias. Third, the relatively small number of participating dental technicians limited statistical power for some analyses and required the use of exact statistical methods because of sparse data. Fourth, communication practices were evaluated through participants’ perceptions rather than objective assessments of laboratory prescriptions, clinical records, or communication exchanges. Finally, patient outcomes and long-term prosthetic performance were not evaluated; therefore, the relationships between decision-making processes, communication practices, and clinical outcomes should be interpreted cautiously. In addition, the use of convenience sampling may limit the representativeness and generalizability of the findings to the wider population of dentists and dental technicians. Most respondent dentists are prosthodontists, and a large proportion completed their postgraduate studies in Saudi Arabia, which may further decrease sample representativeness. I could not calculate both the conventional response rate and potential non-response bias since the questionnaires were distributed through several professional channels. So, the overall count of professionals who received the invitation could not be ascertained. Even though the questionnaires were evaluated by multidisciplinary expert review and were piloted, quantitative content validity assessment and test–retest reliability were not conducted, which must be reflected in the interpretation of the findings of this study. Since all participants were recruited in Saudi Arabia, which make it challenging to generalize these findings to entities with other healthcare systems, educational settings, or clinical–laboratory workflows. Similarly, the high proportion of prosthodontist respondents and the relatively high proportion of Saudi graduates may limit the representativeness of the sample. Future multicenter studies incorporating objective clinical measures and larger sample sizes are warranted to further investigate these relationships.

## 5. Conclusions

Dentists stated that selection of implant abutment was mainly influenced by clinical and prosthetic considerations, specifically implant position and angulation, inter-occlusal space, esthetic requirements, soft tissue thickness, and prosthesis type. Dentists and dental technicians generally favored collaborative decision-making, although communication-related variables were not significantly associated with reported clinical complications. Further studies with larger representative samples and objectively examined clinical outcomes are required to clarify the impact of dentist–technician communication and implant prosthodontic outcomes.

## Figures and Tables

**Table 1:** Characteristics and questionnaire responses of participating dentists (N = 101).

Variable	Category	N	%
**Participant Characteristics** **(N = 101)**			
**Specialty**	Prosthodontist	82	81.2
	Restorative dentist	8	7.9
	AEGD	4	4.0
	Oral surgeon	3	3.0
	Periodontist	2	2.0
	Maxillofacial surgeon	1	1.0
	Resident	1	1.0
**Postgraduate Education**	Saudi Arabia	56	55.4
	North America	26	25.7
	UK and Europe	8	7.9
	Other countries	11	10.9
**Years of Professional Experience**	<5 years	19	18.8
	5.1–10 years	21	20.8
	10.1–15 years	22	21.8
	>15 years	39	38.6
**Practice Setting**	Private	60	59.4
	Academic	39	38.6
	Government	22	21.8
**Communication with Laboratory** **Technicians (Valid N = 101)**			
**I regularly communicate with laboratory technicians regarding abutment selection**	Strongly agree	56	55.4
	Agree	28	27.7
	Neutral	15	14.9
	Disagree	2	2.0
**I provide clear written or verbal instructions to the laboratory**	Strongly agree	59	58.4
	Agree	29	28.7
	Neutral	11	10.9
	Disagree	2	2.0
**Laboratory technicians provide useful** **Feedback**	Strongly agree	36	35.6
	Agree	38	37.6
	Neutral	22	21.8
	Disagree	5	5.0
**Factors influencing implant** **Abutment selection**			
**Implant position and angulation**		98	97.0
**Inter-occlusal space**		83	82.2
**Soft tissue thickness and contour**		79	78.2
**Esthetic requirements**		79	78.2
**Type of prosthesis**		76	75.2
**Availability in clinic/laboratory**		27	26.7
**Cost**		25	24.8
**Familiarity with implant system**		25	24.8
**Implant company recommendations**		16	15.8
**Bone thickness**		1	1.0
**Sources of Knowledge Updates**			
**Courses/workshops**		76	75.2
**Manufacturer representative**		61	60.4
**Colleagues**		52	51.5
**Laboratory technician**		36	35.6
**Social media**		28	27.7
**Research literature**		2	2.0
**I do not seek updates**		2	2.0
**Manufacturer website**		1	1.0
**Company manuals/video channels**		1	1.0
**Difficulty adopting updates because of limited scientific evidence** **(Valid N = 101)**	Strongly agree	11	10.9
	Agree	30	29.7
	Neutral	37	36.6
	Disagree	19	18.8
	Strongly disagree	4	4.0
**Experienced complications due to inappropriate abutment selection** **(Valid N = 101)**	Strongly agree	15	14.9
	Agree	33	32.7
	Neutral	22	21.8
	Disagree	24	23.8
	Strongly disagree	7	6.9
**Reported Complications**			
**Screw loosening**		54	53.5
**Implant–abutment misfit**		34	33.7
**Screw fracture**		28	27.7
**Occlusal problems/improper load distribution**		23	22.8
**Prosthesis fracture/chipping**		23	22.8
**Esthetic dissatisfaction**		21	20.8
**None**		20	19.8
**Peri-implant mucositis**		18	17.8
**Difficulty retrieving/modifying prosthesis**		16	15.8
**Abutment fracture**		13	12.9
**Peri-implantitis**		11	10.9
**Debonding of crown to abutment**		1	1.0
**Responsibility for complications** **(Valid N = 101)**			
**Shared responsibility**		41	40.6
**Clinician**		37	36.6
**Cannot determine/Unsure**		14	13.9
**Laboratory technician**		7	6.9
**Implant manufacturer**		2	2.0

Factors influencing implant abutment selection, sources of knowledge, and reported complications were multiple-response questions; therefore, percentages do not total 100%.

**Table 2:** Characteristics and questionnaire responses of participating dental technicians (N = 43).

Variable	N (%) *
**Participant characteristics (n = 43)**	
**Years of experience in implant-supported prosthetics**	
**<5 years**	17 (39.5)
**5.1–10 years**	8 (18.6)
**10.1–15 years**	6 (14.0)
**>15 years**	12 (27.9)
**Practice setting**	
**Private**	29 (67.4)
**Government**	7 (16.3)
**Academic**	2 (4.7)
**Academic and government**	1 (2.3)
**Private and academic**	2 (4.7)
**Private and government**	2 (4.7)
**Communication practices**	
**Dentists provide clear instructions regarding the type of abutment**	
**Strongly agree**	9 (20.9)
**Agree**	17 (39.5)
**Neutral**	10 (23.3)
**Disagree**	6 (14.0)
**Strongly disagree**	1 (2.3)
**I assist dentists in selecting the appropriate implant abutment**	
**Strongly agree**	18 (41.9)
**Agree**	21 (48.8)
**Neutral**	3 (7.0)
**Disagree**	1 (2.3)
**Problems occur because of poor communication with the dentist**	
**Strongly agree**	15 (34.9)
**Agree**	16 (37.2)
**Neutral**	7 (16.3)
**Disagree**	3 (7.0)
**Strongly disagree**	2 (4.7)
**I initiate communication when the selected abutment is** **Inappropriate**	
**Strongly agree**	26 (60.5)
**Agree**	15 (34.9)
**Disagree**	2 (4.7)
**I prefer collaborative decision-making with the dentist**	
**Strongly agree**	24 (55.8)
**Agree**	16 (37.2)
**Neutral**	3 (7.0)
**Knowledge and professional updates**	
**I keep up to date with new implant abutment** **Systems and components**	
**Strongly agree**	29 (67.4)
**Agree**	7 (16.3)
**Neutral**	7 (16.3)
**I modify laboratory protocols based on new updates**	
**Strongly agree**	25 (58.1)
**Agree**	14 (32.6)
**Neutral**	4 (9.3)
**I trust information from dentists more than from** **Company representatives**	
**Strongly agree**	6 (14.0)
**Agree**	20 (46.5)
**Neutral**	12 (27.9)
**Disagree**	5 (11.6)
**Sources of knowledge updates**	
**Courses/training workshops**	25 (58.1)
**Direct communication with dentists**	22 (51.2)
**Colleagues**	21 (48.8)
**Social media**	16 (37.2)

* Percentages were calculated using the number of valid responses for each questionnaire item. Sources of knowledge were collected using a multiple-response question; therefore, percentages do not total 100%.

**Table 3:** Association between providing clear written or verbal instructions to the laboratory for abutments and experiencing clinical complications due to inappropriate implant abutment selection among dentists (N = 101).

Variable	Statistical Test	Test Statistic	*p* Value
**Providing clear written or verbal instructions to the laboratory vs. experiencing clinical complications**	Fisher–Freeman–Halton exact test *	7.439	0.870

* The Fisher–Freeman–Halton exact test was used because the assumptions of the Pearson chi-square test were violated owing to sparse expected cell counts. Statistical significance was set at *p* < 0.05.

**Table 4:** Association between dental technicians’ perception that dentists provide clear instructions regarding the type of abutment and the perception that problems occur due to poor communication with the dentist (N = 43).

Variable	Statistical Test	Test Statistic	*p* Value
**Dentists provide clear instructions regarding the type of abutment vs. problems occur due to poor communication**	Fisher–Freeman–Halton exact test	16.462	0.420

**Table 5:** Association between dental technicians’ preference for collaborative decision-making and taking the initiative to communicate with the dentist when the selected abutment is considered inappropriate (N = 43).

Variable	Statistical Test	Test Statistic	*p* Value
**Preference for collaborative decision-making vs. taking the initiative to communicate with the dentist**	Fisher–Freeman–Halton exact test	8.391	0.040

**Table 6:** Factors associated with self-reported implant abutment-related complications among clinicians.

Associated Factors	OR	95% CI	*p* Value
**Years of professional experience ^a^**	0.78	0.53–1.15	0.202
**Prosthodontist vs. other specialties**	2.56	0.78–8.39	0.121
**Postgraduate training in Saudi Arabia vs. other countries**	0.80	0.32–2.04	0.647
**Private practice**	1.38	0.53–3.60	0.513
**Academic practice**	0.60	0.24–1.50	0.276
**Government practice**	1.65	0.55–4.96	0.371
**Regular communication with dental technicians ^b^**	0.73	0.43–1.24	0.242

^a^ Years of experience were inserted as an ordinal variable: <5, 5.1–10, 10.1–15, and >15 years. ^b^ Communication was changed to an ordinal Likert variable as well.

## Data Availability

The raw data supporting the conclusions of this article will be made available by the authors on request.

## Supplementary Materials

The following supporting information can be downloaded at: https://www.mdpi.com/article/10.3390/healthcare14172793/s1, Table S1: Descriptive distribution of implant abutment selection factors according to dentists’ communication with laboratory technicians.
