# Supplementary material for: Collaborative Decision-Making in Implant Abutment Selection: Perspectives of Dentists and Dental Technicians in a Cross-Sectional Study
Source: Healthcare (Basel). 2026 Sep 1;14(17):2793. doi: 10.3390/healthcare14172793 (PMC13566594; doi:10.3390/healthcare14172793)
Supplement: Supplementary file 1 [file healthcare-14-02793-s001.zip › healthcare-4492454-Supplementary.pdf]

Table: Supplementary 1. Descriptive distribution of implant abutment selection factors according to dentists' communication with laboratory technicians.

Implant abutment selection factor	Agree n (%) (n=29)	Disagree n (%) (n=2)	Neutral n (%) (n=15)	Strongly agree n (%) (n=56)
**Implant position and angulation**	28 (96.6)	1 (50.0)	15 (100.0)	54 (96.4)
**Inter-occlusal space**	28 (96.6)	1 (50.0)	10 (66.7)	44 (78.6)
**Aesthetic requirements**	25 (86.2)	2 (100.0)	12 (80.0)	40 (71.4)
**Type of prosthesis (single crown, bridge, full-arch)**	22 (75.9)	2 (100.0)	10 (66.7)	42 (75.0)
**Soft tissue thickness and contour**	25 (86.2)	2 (100.0)	11 (73.3)	41 (73.2)
**Availability or stock in clinic/laboratory**	12 (41.4)	0 (0.0)	4 (26.7)	11 (19.6)
**Cost**	10 (34.5)	0 (0.0)	2 (13.3)	13 (23.2)
**Familiarity with implant system/component**	11 (37.9)	0 (0.0)	5 (33.3)	9 (16.1)
**Recommendations from implant company/agent**	5 (17.2)	0 (0.0)	3 (20.0)	8 (14.3)
**Bone thickness**	0 (0.0)	0 (0.0)	0 (0.0)	1 (1.8)

Percentages are calculated within each communication category. Participants could select more than one implant abutment selection factor; therefore, percentages do not sum to 100%. The data are presented descriptively only because this was a multiple-response question.
